# Perspectives on cellular senescence and short term dietary restriction in adults

**DOI:** 10.18632/aging.100247

**Published:** 2010-12-15

**Authors:** James L. Kirkland

**Affiliations:** Robert and Arlene Kogod Center on Aging, Mayo Clinic, Rochester, MN 55905, USA

## INTRODUCTION

Recent evidence supports the contention that cellular senescence is associated with, and may even be a cause of age-related functional impairment. Senescent cells accumulate in multiple tissues with advancing age [1]. Cellular senescence causes a variety of cell types to acquire a pro-inflammatory secretory phenotype that produce a variety of cytokines, chemokines, and extracellular matrix remodeling proteases that are associated with tissue destruction [2-5]. Chronic presence of senescent cells can accelerate cancer progression, possibly because of this inflammatory secretory phenotype [6-7]. Engineered deposition of senescent cells in a single organ, skin, can cause functional impairments in multiple organs similar to those occurring with aging [8]. Finally, senescent cell accumulation in progeroid animal models is associated with dysfunction resembling that of aging [9]. Caloric restriction attenuates processes that have been implicated in cellular senescence, including generation of reactive oxygen species (ROS), growth hormone/insulin-like growth factor-1 signaling, and inflammation [10-12].

*Does caloric restriction in fact reduce cellular senescence?* In an important study in this journal, Wang et al. found short term dietary restriction in middle-aged mice is associated with decreased abundance of senescent cells in the liver (centrilobular hepatocytes) and intestine (crypt enterocytes) [13]. They detected this reduction following 3 months of 26% caloric restriction in 17 month old mice by assaying cellular γH2AX (a protein that associates with damaged DNA), PCNA (an indicator of replication), and senescence-associated β-galactosidase (SA β-gal). The relatively short period of caloric restriction resulted in a 3.3 to 6.5% decrease in senescent cell abundance as a function of total cell number. Despite similar or lower telomerase activity in the calorically restricted compared to *ad libitum*-fed mice, average telomere length in crypt enterocyte nuclei was greater in the calorically restricted animals. The telomere preservation could have been a result of reduced ROS in the calorically restricted animals, since ROS accelerate telomeric DNA loss. Consistent with this possibility, the authors found that markers of cellular oxidative damage (4-HNE and broad-band autofluorescence) were lower in the cell types in which senescence was reduced in the calorically restricted mice. Additional experiments need to be done to test if the association between reduced oxidative damage following short term caloric restriction and decreased senescence reflects a causal relationship. The authors suggest that reduced cellular senescence might be a primary effect of caloric restriction. They speculate this could be mediated through suppression of signaling through mTOR and S6K1, and that this might contribute to improved mitochondrial function and reduced ROS. These possibilities need to be tested directly in future studies.

As with many interesting studies, this study raises many more questions than it answers. Several of these questions, their potential implications, and suggestions for further study follow.

*Does short term caloric restriction reduce the proportion of senescent cells in other tissues, such as fat?* Obesity, aging, and other conditions are associated with extensive accumulation of senescent cells in fat tissue of rodents as well as humans, in whom fat is frequently the most abundant tissue in the body [14-16]. Fat tissue becomes inflamed in association with senescent cell accumulation, potentially leading to clinically important systemic consequences such as diabetes and atherosclerosis. Fat tissue is also among the first tissues to be affected structurally and metabolically by caloric restriction, so determining the impact of short term caloric restriction on fat tissue cellular senescence is particularly important. It would also be interesting to determine the impact of short term caloric restriction on cellular senescence in other tissues, including the brain. This could be particularly instructive in animal models of chronic diseases in which cellular senescence appears to play a role, such as in animal models of progerias or Alzheimer's disease.

*Does short term caloric restriction also reduce abundance of senescent cells and inflammation in humans?* It may not, since, as the authors point out, proliferation-competent skin fibroblasts did not increase after 9 to 12 years of caloric restriction in rhesus monkeys [17]. On the other hand, it might, since white blood cell DNA fragmentation (by comet assay) declines in overweight but non-obese adult human subjects subjected to 25% caloric restriction for 6 months [18]. If sufficient baseline cellular senescence is in fact evident in *ad libitum*-fed subjects in accessible tissues (for example, subcutaneous fat) from subjects in current human trials of short term caloric restriction, it may be informative to determine if decreases in cellular senescence occur in the calorically restricted subjects.

*What is the cell dynamic mechanism that causes decreased senescence during short term caloric restriction?* Altered rates of senescent cell formation or removal could be responsible for the effect of short term caloric restriction on abundance of senescent cells. Senescent cells normally accumulate at a rate of 0.5% or less per month as a function of total cell number in the tissues examined. Caloric restriction resulted in a 3.3 to 6.5% decrease in senescent cell abundance as a function of total cell number. Since senescent cells generally appear to turn over slowly, this implies that declines in production of new senescent cells may be accompanied by increased removal. Senescent cells can be removed through activation of the immune system, with the immune system being activated by factors released by senescent cells [4]. Short term caloric restriction could enhance immune system responsive-ness to senescent cells, a possibility that remains to be tested. Fully established cellular senescence might not be easily reversible, further suggesting that short term caloric restriction enhances clearance of senescent cells by the immune system. Whether caloric restriction improves immune recognition and clearance of senescent cells is an issue that merits further study.

*What is the molecular mechanism behind the decrease in senescent cells due to short term caloric restriction?* This is a key question in determining if there is a path to mechanism-based clinical interventions that would reduce the low grade tissue inflammation associated with many of the diseases common in old age. As the authors state, long term caloric restriction reduces phosphorylation and activity of Akt1, mTOR, and its downstream targets S6K1 and 4E-BP1. Activated S6K1 binds to Mdm2, inhibiting Mdm2-mediated p53 ubiquitination, increasing p53 stability, and thereby potentially inducing senescence. Whether this sequence of events occurs in short term caloric restriction needs to be investigated.

It would be very informative to test if rapamycin or reducing expression of S6K1 decreases senescent cell abundance in centrilobular hepatocyte and intestinal crypt enterocyte populations in a manner similar to short term caloric restriction. It would also be informative to test if expressing activated S6K1 or increasing activity of other mTOR pathway components blocks the impact of caloric restriction on reducing accumulation of senescent cells. Additionally, it would be interesting to test if Akt activation, perhaps by IGF-1, prevents the caloric restriction-related reduction in cellular senescence, especially as IGF-1 decreases within hours to days after starting food restriction in rodents [19-20].

*What are the effects of short compared to long term caloric restriction on cellular senescence and inflammation? How rapidly does the decline in cellular senescence occur and does this vary among tissues?* Although they might appear incremental, time consuming, and expensive, time course studies are necessary. These studies could provide some mechanistic information, but they will be especially valuable when it comes to designing potential interventions.

*Are effects of short term caloric restriction different in old than younger individuals?* The burden of senescent cells increases in most tissues with aging. If short term caloric restriction indeed reduces cellular senescence in a variety of tissues, it would appear quite plausible that the impact of short term caloric restriction would be greater in older than younger individuals. However, macrophage induction by the types of chemokines released by senescent cells may decline with aging [14,21]. If the mechanism of reduced senescent cell abundance is related to increased clearance by activating the immune system, the intervention could actually be less effective in very elderly than middle-aged individuals. This issue will need to be tested experimentally. Furthermore, as the authors point out, late onset caloric restriction could have beneficial effects in humans since even if long term caloric restriction is initiated in later life, there are declines in cancer incidence and enhancements in cognitive function and immune responses. Indeed, the latter could account for the senescent cell clearance induced by caloric restriction.

*Can clinical interventions be developed based on reducing cellular senescence and inflammation through manipulating the pathways activated by short term caloric restriction?* This is of course the most important question. Depending on the answers to the preceding questions, this may be a possibility. Although highly speculative, attacking senescent cells or their pro-inflammatory phenotype could form the basis for developing pharmacological interventions that could reduce age-related frailty, metabolic and cognitive dysfunction, cancers, and other potential consequences of widespread tissue inflammation as a group. Before pursuing this further, it will be necessary to prove that removing senescent cells selectively can restore function in old age and delay age-related disease onset.
